# Improved Fusion of Optical Flow and Dead Reckoning for UAV Navigation Using Digital Terrain Models and Data-Driven Velocity Correction

**DOI:** 10.3390/s26175421

**Published:** 2026-08-27

**Authors:** Jakub Walczak, Piotr Targowski, Szymon Chmielewski, Sebastian Łeska, Janusz Furtak

**Affiliations:** Faculty of Cybernetics, Military University of Technology, 00-908 Warsaw, Poland; piotr.targowski@wat.edu.pl (P.T.); szymon.chmielewski01@wat.edu.pl (S.C.); sebastian.leska@wat.edu.pl (S.Ł.); janusz.furtak@wat.edu.pl (J.F.)

**Keywords:** UAV navigation, optical flow, GPS denied, digital terrain model, dead reckoning, Kalman filter, data-driven correction, velocity estimation, Farnebäck algorithm, PX4

## Abstract

Accurate velocity estimation in GPS-denied environments remains a core challenge for autonomous unmanned aerial vehicle (UAV) navigation. Our previous work demonstrated that fusing optical flow (OF) with dead reckoning (DR) substantially reduces position drift compared to inertial-only solutions. However, velocity estimates derived from dense optical flow are degraded by two systematic effects: (1) incorrect metric scaling when the camera footprint covers heterogeneous terrain types—particularly at forest–field transitions where the visible surface elevation differs significantly from bare-ground elevation; and (2) flow magnitude bias introduced by scene texture and structural properties. This paper presents three targeted improvements to a software pipeline for optical flow-assisted UAV navigation. First, single-point above-ground-level (AGL) estimation is replaced by camera footprint area mean sampling over co-registered Digital Terrain Model (DTM) and Digital Surface Model (DSM) rasters, with the surface model adopted consistently for OF metric scaling. Second, a one-dimensional Kalman filter with an innovation gate suppresses velocity spikes caused by abrupt terrain transitions. Third, a compact data-driven correction module uses selected flow, texture, and terrain descriptors to estimate a multiplicative velocity correction factor aligned with GPS-derived reference speed available during calibration and offline evaluation but not required during GPS-denied operation. Experiments on three real PX4-logged flight missions (Log 258 for calibration and Logs 259–260 for independent evaluation) totalling 7.3 min show terrain-dependent behaviour. On the mixed forest–field validation flight (Log 260), the improved pipeline reduces velocity mean absolute error (MAE) by 71% (1.04 m/s → 0.30 m/s) and dead-reckoning position MAE by 88% (59.8 m → 7.1 m), compared to the baseline from our previous work. On a flat open-terrain validation flight (Log 259), the terrain-aware modifications leave the terrain-insensitive baseline essentially unchanged, providing a control case for the proposed DSM-based scaling mechanism.

## 1. Introduction

Autonomous unmanned aerial vehicles (UAVs) operating in GPS-denied or GPS-degraded environments must rely on alternative navigation strategies to maintain positional awareness. Dead reckoning (DR)—the incremental integration of velocity and track angle—provides a continuous navigation solution independent of external infrastructure, but it accumulates error over time due to sensor noise and drift [1]. Optical flow (OF), the apparent motion of image features across successive camera frames, offers a complementary velocity measurement that does not integrate over time and therefore does not exhibit secular drift [2].

Our earlier work [3] demonstrated a practical fusion architecture for low-altitude UAV navigation in which dense Farnebäck optical flow computed from a nadir-pointing camera is combined with DR to suppress position error in GPS-denied conditions. The baseline approach estimated ground-plane velocity from optical flow magnitude, camera geometry, and above-ground-level (AGL) altitude, and then integrated this velocity estimate within a dead-reckoning module. Although the results were promising, subsequent analysis revealed two systematic limitations that motivate the present work.

**Terrain heterogeneity.** Low-altitude flights over mixed terrain—common in reconnaissance and precision agriculture missions—frequently cross forest–field boundaries. Over forested areas, the camera tracks the top-of-canopy surface rather than bare ground. If the AGL altitude is computed using a Digital Terrain Model (DTM, bare-earth elevation), the derived pixel scale underestimates the true camera height above the visible surface, causing the velocity estimate to be inflated by up to 40% at sites with 15 m-high canopy at 50 m AGL. Single-point altitude sampling further amplifies boundary artefacts when the terrain type changes rapidly within the camera footprint.

**Flow magnitude bias.** The flow magnitude is sensitive to scene texture, motion blur, and illumination gradients unrelated to camera velocity. Smoother textures (e.g., uniform crop fields) produce systematically lower flow magnitudes for the same ground speed than high-frequency textures (e.g., forest canopy), introducing a scene-dependent offset that a purely geometric model cannot correct.

This paper presents three targeted improvements to the previously reported processing pipeline:

1. **Camera footprint area mean terrain sampling.** The camera ground footprint is approximated from altitude and camera geometry and sampled from both DTM and DSM rasters. The surface model mean (${\bar{h}}_{\mathrm{surf}}$) is used for OF metric scaling.
2. **Kalman filter with innovation gate.** A one-dimensional Kalman filter with bounded innovations is applied to the velocity estimate, suppressing impulsive spikes without sacrificing tracking responsiveness.
3. **Data-driven velocity correction.** A compact regression module maps selected flow, texture, and terrain descriptors to a scalar correction factor α learned from real flight data.

For readability, the principal abbreviations used throughout the paper are introduced here: optical flow (OF), dead reckoning (DR), above-ground level (AGL), Digital Terrain Model (DTM), Digital Surface Model (DSM), Global Positioning System (GPS), and unmanned aerial vehicle (UAV). A complete abbreviation list is also provided at the end of the manuscript.

The main physical mechanism can be understood from the pinhole-camera scale relation

(1) $$s=\frac{hp}{f},$$

where *s* is the ground sampling scale in m/pixel, *h* is the distance from the camera to the visible surface, *p* is the pixel pitch, and *f* is the focal length. Equivalently, the implementation uses the diagonal field of view to express the same scale without separately requiring *p* and *f* (Section 4.1). An error δh in the assumed visible surface height produces an approximately proportional velocity-scale error δh/h. Thus, the terrain-aware part of the pipeline addresses the primary metric-scaling error, whereas the Kalman gate and the data-driven correction provide robustness against transition spikes and residual optical flow bias.

The remainder of the paper is structured as follows. Section 2 reviews related work. Section 3 describes the software processing architecture. Section 4 details the algorithmic contributions. Section 5 presents experimental results. Section 6 discusses limitations and future directions, and Section 7 concludes the paper.

## 2. Related Work

Optical flow for ego-motion estimation has a long history in robotics. Horn and Schunck [4] and Lucas and Kanade [5] established the two principal paradigms—global and local OF estimation. Farnebäck’s dense polynomial expansion method [6] remains widely used in UAV applications due to its robustness and availability in OpenCV [7]. Recent learning-based optical flow and visual-correspondence methods, including PWC-Net, RAFT, LoFTR, and DROID-SLAM [8,9,10,11], have substantially improved benchmark accuracy and robustness in complex visual scenes. In contrast, the present implementation deliberately uses a classical dense OF estimator because it is deterministic, lightweight, and directly interpretable in terms of metric scale errors. Modifications to improve robustness under partial occlusion and motion blur have been proposed for fixed-wing platforms [12], but the baseline algorithm performs well for quasi-hovering rotary-wing UAVs at moderate altitudes.

Terrain-referenced navigation for fixed-wing aircraft uses digital elevation models to correct barometric altitude [13]. Similar ideas have been applied to rotary-wing UAVs for altitude hold in GPS-denied environments [12]. The use of co-registered DTM/DSM pairs to resolve the bare-ground vs. top-of-canopy ambiguity in OF metric scaling has not, to the best of the authors’ knowledge, been previously reported.

Statistical characterisation of the optical flow field beyond mean magnitude has been used for obstacle detection [2] and landing zone assessment [14], but not for velocity estimation accuracy improvement. Machine learning corrections for sensor fusion have been proposed in the context of inertial navigation [15], while modern visual-inertial systems such as VINS-Mono and event-camera odometry demonstrate the value of tightly coupled visual and inertial estimation for GPS-degraded operation [16,17]. Our approach targets a specific, interpretable error mechanism (terrain-induced scale bias) rather than learning a general odometry correction end-to-end.

## 3. Software Processing Architecture

### 3.1. Overview

The evaluated implementation is an offline C++ processing pipeline using OpenCV 4.5.4 and GDAL 3.4.1 [18]. It ingests files recorded during a single UAV mission and produces tabular outputs containing per-frame velocity and dead-reckoning position estimates alongside GPS-derived reference values used for offline validation. The implementation is designed for repeatable ground-station post-processing and experimental comparison rather than for disclosure of a production runtime architecture.

**Figure 1:**
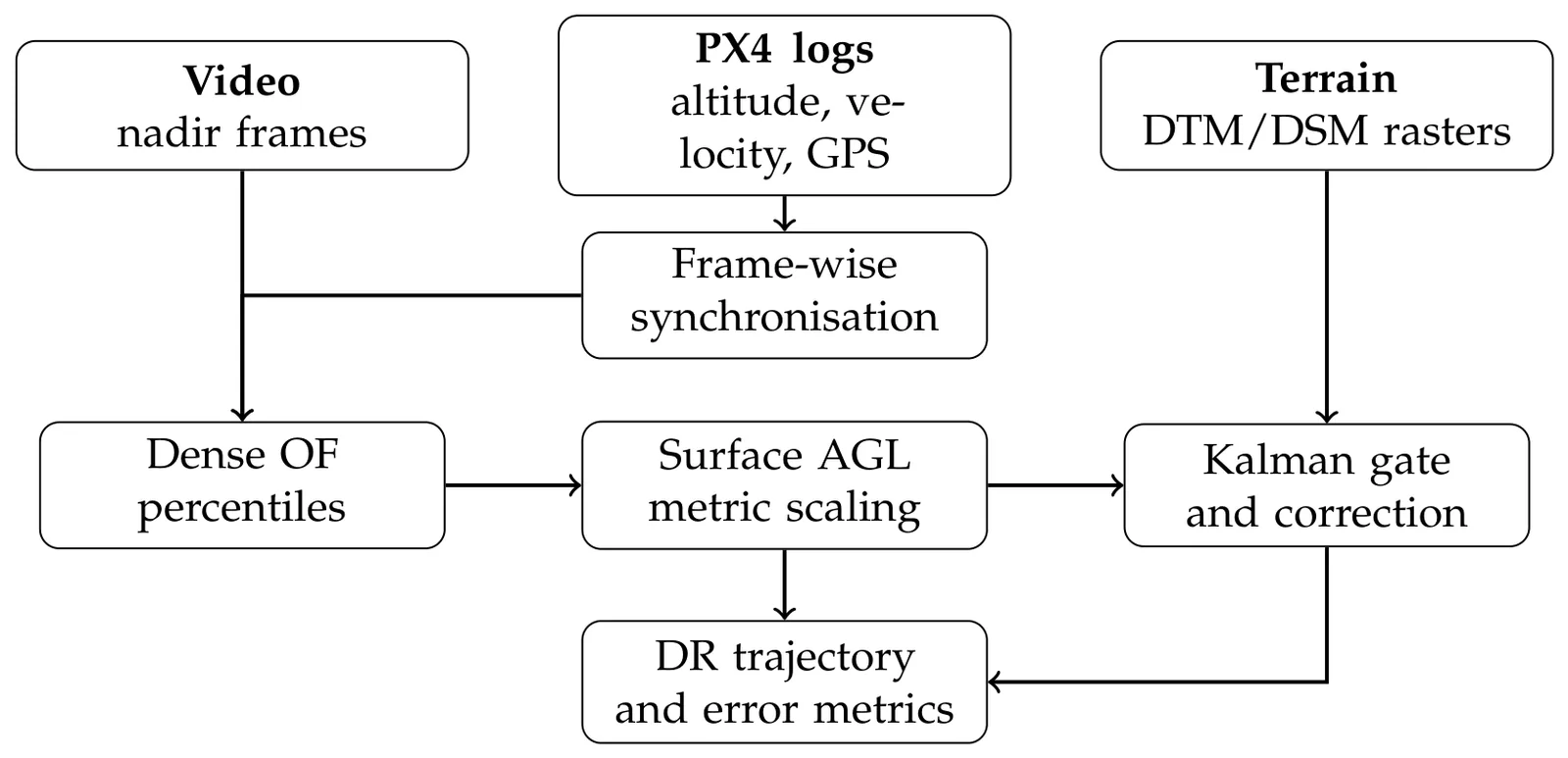
Signal-flow sketch of the evaluated processing pipeline. GPS is used only as reference data for calibration and offline evaluation, not as an input in GPS-denied operation.

Figure 1 summarises the signal flow. The video stream provides OF statistics and texture descriptors, PX4 logs provide barometric altitude, track angle, and GPS reference values, and the DTM/DSM rasters provide the terrain-aware surface height used for metric scaling. These streams are synchronised per processed frame before velocity filtering, bounded data-driven correction, and dead-reckoning integration.

### 3.2. Input Data

The processing pipeline consumes three input streams from a single mission directory:

**Flight computer logs.** The primary flight data is exported from PX4 [19] autopilot log files using ulog2csv from the pyulog package, version 1.2.2. Two CSV files are used:

*_vehicle_local_position_0.csv—contains local-frame horizontal velocity components ($v_{x}$, $v_{y}$, m/s), barometric altitude *z* (m, North–East–Down (NED) convention, positive down), and microsecond-resolution timestamps. Here, “local-frame” denotes the navigation frame exported by PX4 rather than the UAV body frame. The horizontal speed and track angle are derived as $v=\sqrt{v_{x}^{2}+v_{y}^{2}}$ and $\psi =\mathrm{atan}2(v_{x},v_{y})$, respectively, following the axis convention used in the exported logs.
*_vehicle_gps_position_0.csv—provides WGS-84 latitude and longitude (integer $\times 10^{-7}$ deg) and GPS speed-over-ground vel_m_s used as the offline velocity reference for calibration and evaluation. This signal is not an input to the navigation estimate in simulated GPS-denied operation.

**Video file.** A nadir-pointing camera records a downward-facing video in H.264/MP4 format. OpenCV’s VideoCapture is used to read frames sequentially; a configurable frame-skip parameter *S* (command line flag -S) allows processing every *S*-th frame, with intermediate frames discarded via cap.grab() to advance the decoder state.

The camera model used in the metric conversion is summarised in Table 1. The implementation uses an equivalent pinhole/FOV model; therefore, pixel pitch and physical focal length are not required separately as long as the effective field of view and image dimensions are known. The exported flight/video dataset did not contain frame-level exposure time or shutter readout metadata, and no separate laboratory intrinsic calibration was available for these logs. Radial distortion is assumed to be small after gimbal stabilisation and central-frame processing; this assumption should be revisited in deployments using wide-angle or non-stabilised cameras.

**Terrain rasters.** Two single-band GeoTIFF rasters in the EPSG:2180 Polish national grid projection:

- terrain_model/DTM.tiff—bare-earth Digital Terrain Model (DTM).
- terrain_model/DSM.tiff—Digital Surface Model (DSM) including vegetation and buildings.

Rasters are loaded via cv::imread(IMREAD_UNCHANGED) and georeferenced using gdalinfo output parsed at startup. Coordinate transformation from WGS-84 (EPSG:4326) to the raster coordinate reference system (CRS) is performed on-the-fly via gdaltransform.

### 3.3. Stream Synchronisation

The three input streams operate at different sampling frequencies. Video is recorded at 30 fps (configurable, -F flag). The vehicle local position log typically runs at ∼50 Hz, and the GPS log at ∼5 Hz. To align them without resampling, the software estimates the actual frequency of each CSV by computing the mean inter-sample interval from the first 100 timestamp entries. An advance counter drives how often each stream is advanced per processed frame:

(2) $$N_{\log}=\left\lfloor f_{\mathrm{eff}}/f_{\log}\right\rceil ,\;N_{\mathrm{GPS}}=\left\lfloor f_{\mathrm{eff}}/f_{\mathrm{GPS}}\right\rceil ,$$

where $f_{\mathrm{eff}}=f_{\mathrm{video}}/S$ is the effective frame rate after skip. One log row is consumed every $N_{\log}$ video frames, and one GPS row every $N_{\mathrm{GPS}}$ video frames, maintaining temporal alignment without interpolation. This procedure assumes that residual timestamp offsets between the video, local-position, and GPS streams are small relative to the vehicle dynamics and the 30 fps video period. Such offsets are not explicitly estimated in the present implementation; their effect is treated as part of the measurement error absorbed by the Kalman measurement noise parameter and by the offline comparison against GPS.

### 3.4. Processing Loop

The main loop iterates over the longest available stream. At each iteration:

1. Advance log and GPS streams per the counters in Equation (2).
2. Decode the next video frame (skipping S−1 intermediate frames).
3. Compute the camera footprint and sample terrain models (Section 4.2).
4. Update the optical flow processor; extract FlowStats and texture variance (Section 4.1).
5. Apply the Kalman filter and optionally the data-driven correction (Section 4.3 and Section 4.4).
6. Feed the corrected speed, barometric altitude, and track angle to the dead reckoning module (Section 4.5).
7. Write one CSV row to the output file.

### 3.5. Output Format

Table 2 defines the 23-column CSV row produced for each processed frame:

## 4. Algorithms

### 4.1. Optical Flow Velocity Estimation

For each pair of consecutive grayscale frames, the dense optical flow field $F\in R^{W_{p}\times H_{p}\times 2}$ is computed using cv::calcOpticalFlowFarneback with the parameter set listed in Table 3. Frames are downsampled to a processing height $H_{p}$ (default 360 px, configurable via -P) while preserving the aspect ratio; $W_{p}$ and $H_{p}$ denote the processing frame width and height, respectively. This reduces computation time roughly quadratically with the downscale factor.

For pixel indices *i* and *j* in the processing image, the per-pixel magnitude $m_{i,j}={∥F_{i,j}∥}_{2}$ is computed via cv::magnitude. All $W_{p}\cdot H_{p}$ values are sorted and five percentiles (P10, P25, P50, P75, P90) are extracted. The median P50 serves as the primary velocity proxy; the full distribution is passed to the data-driven correction module. Directional coherence is

(3) $$C=\min\left(1,\;\frac{\sqrt{{\bar{F}}_{x}^{2}+{\bar{F}}_{y}^{2}}}{\bar{m}}\right),$$

where ${\bar{F}}_{x},{\bar{F}}_{y}$ are the mean flow components and $\bar{m}$ is the mean magnitude. C≈1 indicates pure translation; C→0 indicates rotation or disordered flow.

The metric scale converts flow in px/frame to m/s:

(4) $$v_{\mathrm{OF}}=\underset{\mathrm{px}/\mathrm{diag}}{\underset{︸}{\frac{m_{P50}}{d_{p}}}}\cdot \underset{\mathrm{diag}\;\mathrm{in}\;\mathrm{metres}}{\underset{︸}{\frac{2h_{\mathrm{agl}}\tan\left({\mathrm{FOV}}_{d}/2\right)}{1}}}\cdot f_{\mathrm{eff}},$$

where $d_{p}=\sqrt{W_{p}^{2}+H_{p}^{2}}$ is the processing resolution diagonal in pixels, ${\mathrm{FOV}}_{d}$ is the diagonal field of view in radians, and $h_{\mathrm{agl}}$ is the AGL height selected for metric scaling; in the improved pipeline $h_{\mathrm{agl}}=h_{\mathrm{surf}}$. The focal length in pixels is $f_{\mathrm{px}}=d_{\mathrm{full}}/\left(2\tan\left({\mathrm{FOV}}_{d}/2\right)\right)$, with $d_{\mathrm{full}}$ the full-resolution diagonal, and scales consistently with $H_{p}$.

### 4.2. Terrain-Aware AGL Estimation

The terrain-aware AGL computation distinguishes three height concepts. The barometric altitude $z_{\mathrm{baro}}$ is measured relative to take-off and describes the UAV’s vertical motion. The DTM elevation represents the bare-earth terrain height, whereas the DSM elevation represents the highest visible surface, including vegetation and buildings. Optical flow is generated by the visible surface rather than by the hidden bare-earth surface; therefore, $h_{\mathrm{surf}}$ is the physically relevant scale height for Equation (4). The height quantities used throughout this section are summarised in Table 4.

#### 4.2.1. Raster Loading

At startup, both rasters are loaded into memory as OpenCV cv::Mat objects with depth preserved (float32/float64/uint16/int16 depending on the GeoTIFF encoding). Georeferencing metadata (origin, pixel size, nodata value, and EPSG code) is parsed from the output of gdalinfo using regular expressions. The geotransform matrix

(5) $$\left(\begin{array}{c} x\\ y \end{array}\right)=\left(\begin{array}{c} x_{0}\\ y_{0} \end{array}\right)+\left(\begin{array}{cc} \Delta x & 0\\ 0 & \Delta y \end{array}\right)\left(\begin{array}{c} \mathrm{col}\\ \mathrm{row} \end{array}\right)$$

is stored for fast pixel-coordinate conversion, where $\left(x_{0},y_{0}\right)$ is the raster origin in EPSG:2180 metres and (Δx,Δy) are signed pixel sizes (typically Δx=+1, Δy=−1 m/px at 1 m GSD). The variables col and row denote raster column and row indices.

#### 4.2.2. Camera Footprint Area Sampling

The diagonal FOV is decomposed into horizontal and vertical components. Here, *W* and *H* denote the full-resolution image width and height, while $W_{p}$ and $H_{p}$ are used only after downsampling for OF processing:

(6) $$\theta_{H}=2\arctan\left(\frac{W}{d_{\mathrm{full}}}\tan\frac{{\mathrm{FOV}}_{d}}{2}\right),\;\theta_{V}=2\arctan\left(\frac{H}{d_{\mathrm{full}}}\tan\frac{{\mathrm{FOV}}_{d}}{2}\right).$$

At barometric altitude $z_{\mathrm{baro}}$, the approximate footprint half-widths are

(7) $$r_{x}=z_{\mathrm{baro}}\tan\frac{\theta_{H}}{2},\;r_{y}=z_{\mathrm{baro}}\tan\frac{\theta_{V}}{2}.$$

A 5×5 regular grid spans $\left[-r_{x},r_{x}\right]\times \left[-r_{y},r_{y}\right]$ in metric offsets from the current GPS position. The centre point is transformed from WGS-84 to EPSG:2180 in a single gdaltransform subprocess call. Each of the 25 metric offsets is then applied directly in raster-pixel space:

(8) $${\mathrm{col}}_{\ell }=\left\lfloor \frac{\left(c_{x}+\delta x_{\ell }\right)-x_{0}}{\Delta x}\right\rfloor ,\;{\mathrm{row}}_{\ell }=\left\lfloor \frac{\left(c_{y}+\delta y_{\ell }\right)-y_{0}}{\Delta y}\right\rfloor ,$$

where $\left(c_{x},c_{y}\right)$ is the projected centre, and *ℓ* indexes the 25 footprint samples with metric offsets $\left(\delta x_{\ell },\delta y_{\ell }\right)$. Valid (in-bounds, non-nodata) samples are averaged; the call returns false only if no valid sample exists.

#### 4.2.3. AGL Computation

At the first frame with valid terrain data the take-off reference elevations $h_{\mathrm{DTM}}^{0},h_{\mathrm{DSM}}^{0}$ are recorded. For each subsequent frame, the terrain elevation change relative to the take-off footprint is subtracted from the barometric altitude. If the UAV flies at constant barometric height but the visible surface rises because of trees, $h_{\mathrm{surf}}$ decreases accordingly; this is the correction required by the scale relation in Equation (1). For each subsequent frame:

(9) $$h_{\mathrm{gnd}}=\max\left(1,\;z_{\mathrm{baro}}-\left({\bar{h}}_{\mathrm{DTM}}-h_{\mathrm{DTM}}^{0}\right)\right),$$

(10) $$h_{\mathrm{surf}}=\max\left(1,\;z_{\mathrm{baro}}-\left({\bar{h}}_{\mathrm{DSM}}-h_{\mathrm{DSM}}^{0}\right)\right).$$

The vegetation height estimate is $\Delta h_{\mathrm{veg}}=h_{\mathrm{gnd}}-h_{\mathrm{surf}}$ (positive over forest, zero over bare ground). *Only* $h_{\mathrm{surf}}$ is passed to Equation (4), because the camera always observes the top-of-canopy surface modelled by the DSM. If either raster is unavailable, the raw barometric altitude is used and the output is tagged alt_only.

### 4.3. Kalman Filter with Innovation Gate

The Kalman stage is a causal one-dimensional filter, not a forward–backward smoother. The state is the scalar horizontal speed $v_{k}$ at processed video frame/time step *k*. The underlying random walk state-space model is

(11) $$v_{k}=v_{k-1}+w_{k},\;z_{k}=v_{\mathrm{OF},k}=v_{k}+n_{k},$$

where $w_{k}∼N\left(0,q\right)$ is the process noise and $n_{k}∼N\left(0,r\right)$ is the optical flow measurement noise. The filter is initialised with ${\hat{v}}_{0}=0$ and $P_{0}=1$. The prediction is

(12) $${\hat{v}}_{k}^{-}={\hat{v}}_{k-1},\;P_{k}^{-}=P_{k-1}+q.$$

To avoid ambiguous “clip” notation, the innovation gate is written as the saturation function

(13) $${\mathrm{sat}}_{\gamma }\left(e\right)=\left\{\begin{array}{cc} -\gamma , & e<-\gamma ,\\ e, & -\gamma \leq e\leq \gamma ,\\ \gamma , & e>\gamma , \end{array}\right.$$

with innovation $e_{k}=z_{k}-{\hat{v}}_{k}^{-}$. The update is

(14) $$K_{k}=\frac{P_{k}^{-}}{P_{k}^{-}+r},\;{\hat{v}}_{k}={\hat{v}}_{k}^{-}+K_{k}{\mathrm{sat}}_{\gamma }\left(e_{k}\right),\;P_{k}=\left(1-K_{k}\right)P_{k}^{-}.$$

Parameters are q=0.01 (m/s)^2^/frame, r=2.0 (m/s)^2^, and γ=4.0 m/s. The symbol γ denotes the innovation gate threshold and is used instead of *g* to avoid confusion with gravitational acceleration. The innovation gate bounds impulsive residuals before the Kalman gain is applied. Unlike hard rejection, the saturated residual still moves the estimate toward the measurement, so genuine velocity steps are tracked (just more slowly), while pathological spikes (>γ) cannot shift the state by more than $K_{k}\cdot \gamma$ in one step.

### 4.4. Data-Driven Velocity Correction

#### 4.4.1. Feature Vector

After each Kalman update, the correction stage receives a compact descriptor vector composed of optical flow magnitude percentiles, directional coherence, image texture statistics, and terrain height descriptors:

(15) $$x={\left[v_{P10},\;v_{P25},\;v_{P50},\;v_{P75},\;v_{P90},\;C,\;\sigma_{\mathrm{tex}},\;h_{\mathrm{surf}},\;h_{\mathrm{gnd}},\;\Delta h_{\mathrm{veg}}\right]}^{⊤}.$$

Here, $v_{P\ell }$ denotes the *ℓ*-th optical flow magnitude percentile scaled to m/s (ℓ∈{10,25,50,75,90}), *C* is the directional coherence, $\sigma_{\mathrm{tex}}$ is the grayscale texture statistic, and $\Delta h_{\mathrm{veg}}$ describes the difference between bare-earth and surface model AGL. Near-stationary frames are excluded from the supervised calibration set to avoid unstable target ratios and to focus the correction on translational flight segments.

#### 4.4.2. Correction Target and Calibration

The supervised target is a bounded multiplicative factor,

(16) $$\alpha =\Pi_{\left[\alpha_{\min},\alpha_{\max}\right]}\left(\frac{v_{\mathrm{GPS}}}{v_{\mathrm{OF}}+\varepsilon }\right),$$

where $v_{\mathrm{GPS}}$ is the GPS reference speed used during calibration/evaluation, ε prevents division by zero, and $\alpha_{\min}$ and $\alpha_{\max}$ are the lower and upper admissible correction-factor bounds. The projection operator is

(17) $$\Pi_{\left[a,b\right]}\left(x\right)=\left\{\begin{array}{cc} a, & x<a,\\ x, & a\leq x\leq b,\\ b, & x>b. \end{array}\right.$$

The projection interval bounds frames in which the optical flow estimate fails catastrophically, while preserving the normal range of multiplicative scene-dependent bias.

The correction module is implemented as a fully connected multilayer perceptron regressor in scikit-learn 1.7.2 [20] under Python 3.10.12. The ten features in Equation (15) are standardised using the mean and standard deviation fitted on the calibration subset only. The regressor contains three hidden layers with 64, 32, and 16 neurons, respectively, rectified-linear activations, and a linear scalar output for $\hat{\alpha }$. Training uses the Adam optimiser, $L_{2}$ penalty $\alpha_{L2}=10^{-4}$, initial learning rate $10^{-3}$, maximum 500 epochs, early stopping with a 10% internal validation fraction, patience of 20 epochs, and fixed random seed 42. The target factor is projected to $\alpha_{\min}=0.5$ and $\alpha_{\max}=1.5$; frames with $v_{\mathrm{GPS}}\leq 0.1$ m/s are excluded from calibration. Log 258 is used only for fitting and internal diagnostic validation. Logs 259 and 260 are not used during training and are retained for independent assessment of the complete pipeline.

The important methodological point is that a bounded scalar correction is estimated from physically interpretable per-frame descriptors rather than from raw images or opaque end-to-end odometry. The corrected velocity is computed as

(18) $$\hat{v}=\Pi_{\left[\alpha_{\min},\alpha_{\max}\right]}\left(\hat{\alpha }\right)\cdot {\hat{v}}_{\mathrm{Kalman}}.$$

The correction stage is computationally negligible relative to dense optical flow extraction.

### 4.5. Dead Reckoning Integration

The corrected per-frame speed $\hat{v}$ is fed into the dead reckoning module together with the local-frame track angle ψ (computed from PX4’s $v_{x}$, $v_{y}$ as $\psi =\mathrm{atan}2(v_{x},v_{y})$) and the time step $\Delta t=1/f_{\mathrm{eff}}$. The output trajectory is represented in a local East-North-Up (ENU) frame. ENU is used only for reporting horizontal displacement; the NED convention mentioned earlier refers to the sign convention of the PX4 local-position log. The ENU position is updated as

(19) $$\left(\begin{array}{c} \Delta E\\ \Delta N \end{array}\right)=\hat{v}\Delta t\left(\begin{array}{c} \sin\psi \\ \cos\psi \end{array}\right),$$

and accumulated from the first GPS fix. The accumulated ENU displacement is converted to geodetic latitude/longitude for output. The dead reckoning module is re-seeded (reset to zero ENU) at every GPS sample to limit long-term drift accumulation, making the trajectory error observable per GPS interval rather than globally accumulative.

## 5. Experimental Results

### 5.1. Experimental Setup

Experiments were conducted on three flight logs recorded over a mixed agricultural–forest area near Warsaw, Poland (52.33° N, 21.07° E), using a Yuneec H520 (Yuneec International, Kunshan, China) equipped with a nadir-facing camera (91° diagonal FOV, 1920 × 1080 px, 30 fps). Log 258 was used for calibration of the data-driven correction stage, while Logs 259 and 260 were kept as independent validation flights. Flight parameters and terrain characteristics are summarised in Table 5.

Terrain rasters at 1 m GSD (DTM and DSM) were obtained from the Head Office of Geodesy and Cartography (GUGiK), Poland. All runs used processing height $H_{p}=360$ px and frame skip S=1. The DSM surface model was used in >99.97% of frames in all logs (3883/3884, 3617/3618, and 5710/5711 respectively); one initial frame per log reverts to alt_only before the terrain origin is initialised.

### 5.2. Comparison with Baseline System

Table 6 presents the full comparison between the baseline system from [3] and the improved processing pipeline on all three flight logs. The baseline used mean optical flow magnitude with single-point DTM-based AGL and no Kalman filtering. The improved configuration uses median (P50) magnitude, DSM camera footprint area mean AGL, Kalman filtering with innovation gating, and the data-driven correction stage.

The improvement is substantially larger on Log 260, which contains significant forest cover. On Log 258 and Log 259 (essentially flat terrain with vegetation height <0.25 m throughout the flights), the DSM and DTM rasters are nearly identical; consequently, the terrain-aware component is not expected to provide a substantial geometric correction. Log 259 is therefore a held-out control case and confirms that the proposed DSM-based scaling does not artificially improve all flights. On Log 260, the terrain model contributes directly: the 16.65 m canopy at ∼50 m AGL corresponds to a 33% pixel-scale error in the baseline, which the DSM-based AGL eliminates.

### 5.3. Statistical Treatment of Velocity Errors

To address the statistical reliability of the reported velocity improvements, a paired non-parametric sign test was applied to per-frame absolute velocity errors, using only frames with $v_{\mathrm{GPS}}>0.1$ m/s. The test compares whether the improved pipeline produces a smaller absolute error than the baseline for the same frame. Because adjacent video frames are temporally correlated, the resulting *p*-values should be interpreted as supporting evidence rather than as fully independent-sample flight-level statistics. The analysis nevertheless provides a useful within-log consistency check. Results are summarised in Table 7.

### 5.4. Component-Level Interpretation

The strongest improvement occurs on Log 260, where the DSM–DTM separation reaches 16.65 m. This observation is consistent with the expected geometric scale correction: at approximately 50 m AGL, using bare-earth DTM altitude instead of DSM surface altitude can introduce a pixel-scale error of about one third. By contrast, Log 259 has negligible DSM–DTM separation and does not improve after applying the terrain-aware pipeline (Table 6). This negative control supports the interpretation that the method primarily corrects surface-height-induced scale error rather than uniformly reducing all velocity estimates. The Kalman innovation gate then limits residual transition spikes, while the data-driven module acts as a bounded texture-dependent refinement.

### 5.5. Data-Driven Correction Performance

The correction module was calibrated on Log 258. A held-out split from Log 258 is reported only as an internal calibration diagnostic; it is not used as evidence of cross-flight generalisation. On this diagnostic split, the correction factor estimator achieved MAE =0.036 and the dimensionless coefficient of determination $R^{2}=0.775$, indicating that the selected flow, texture, and terrain descriptors explain a substantial fraction of the residual multiplicative bias within the calibration flight.

Generalisation was assessed using complete flight logs that were not used during training. Log 259 is a flat open-terrain validation case and shows essentially unchanged velocity and position errors (Table 6), which is consistent with the absence of DSM–DTM height separation. Log 260 is the mixed forest–field validation case; its large improvement should therefore be attributed primarily to DSM-based geometric scaling and innovation-gated filtering, with the learned component providing only an additional scene-dependent refinement.

### 5.6. Directional Coherence Analysis

**Figure 2:**
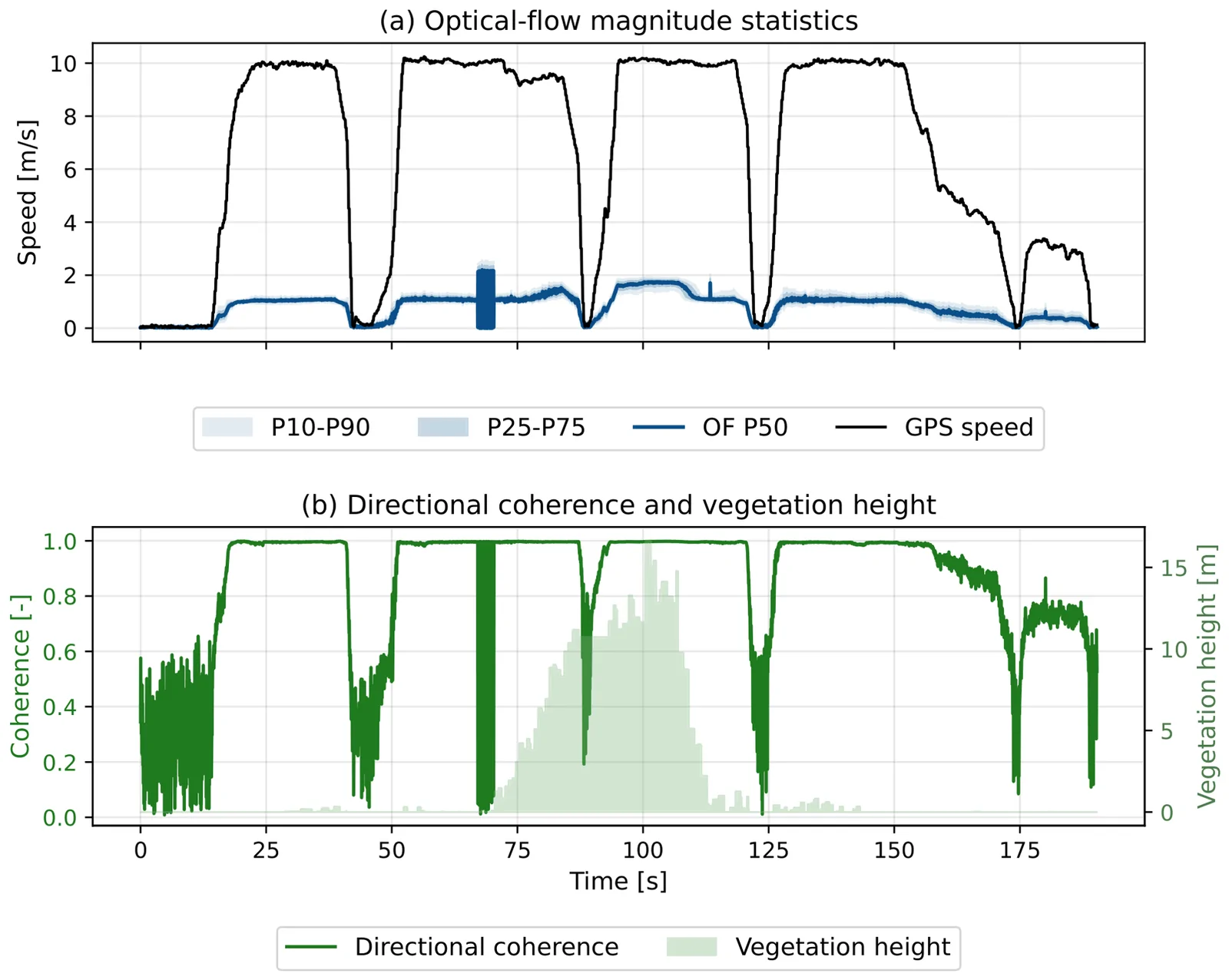
Optical flow field statistics for Log 260 (forest–field flight). (**a**) Flow magnitude percentile bands (P10–P90) compared to GPS ground speed. The median P50 (solid blue) tracks GPS closely; the wide P10–P90 band reflects texture variability across terrain types. (**b**) Directional coherence *C* and vegetation height. Coherence drops during forest overpasses (high vegetation height, green shading), where canopy parallax introduces a non-translational flow component.

Figure 2 and Table 8 report the directional coherence *C* (Equation (3)) across the representative calibration and forest–field validation logs. High coherence (C>0.8) throughout the representative missions confirms that the dominant image motion is translational, validating the single-magnitude velocity proxy. The slightly lower coherence mean on Log 260 (0.846 vs. 0.871) reflects yaw manoeuvres over the forest, where canopy parallax introduces a non-translational flow component. Figure 2b shows the coherence dipping visibly during forest segments (green shading), corroborating the terrain analysis.

### 5.7. Correction Factor Distribution

**Figure 3:**
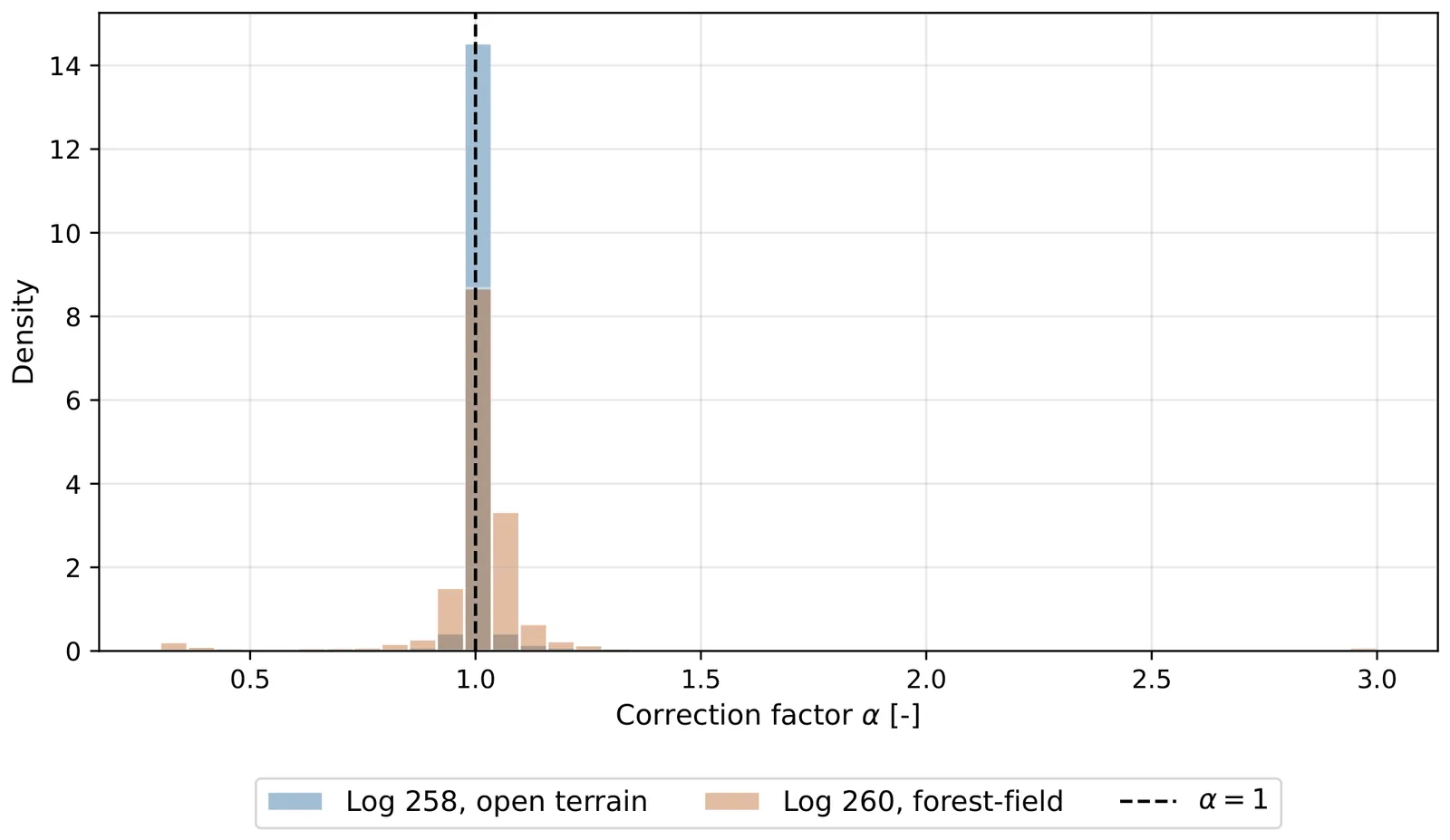
Distribution of the correction factor $\alpha =v_{\mathrm{GPS}}/v_{\mathrm{OF}}$ across the representative Log 258 and Log 260 flights. Left tail (α<0.7): OF overestimation during forest segments (Log 260, $\Delta h_{\mathrm{veg}}$ up to 16.65 m). Right tail (α>1.3): OF underestimation over smooth crop fields.

Figure 3 shows the distribution of α (Equation (16)) over the representative Log 258 and Log 260 flights. The bulk of frames cluster near α=1.0 (well-calibrated OF after DSM correction). The left tail (α<0.7) occurs predominantly on Log 260 during forest overpasses (high $\Delta h_{\mathrm{veg}}$), where even DSM-based scaling slightly overestimates the surface-relative height. The right tail (α>1.3) corresponds to smooth crop fields where low-texture surfaces produce systematically underestimated flow magnitudes.

### 5.8. Velocity Tracking

**Figure 4:**
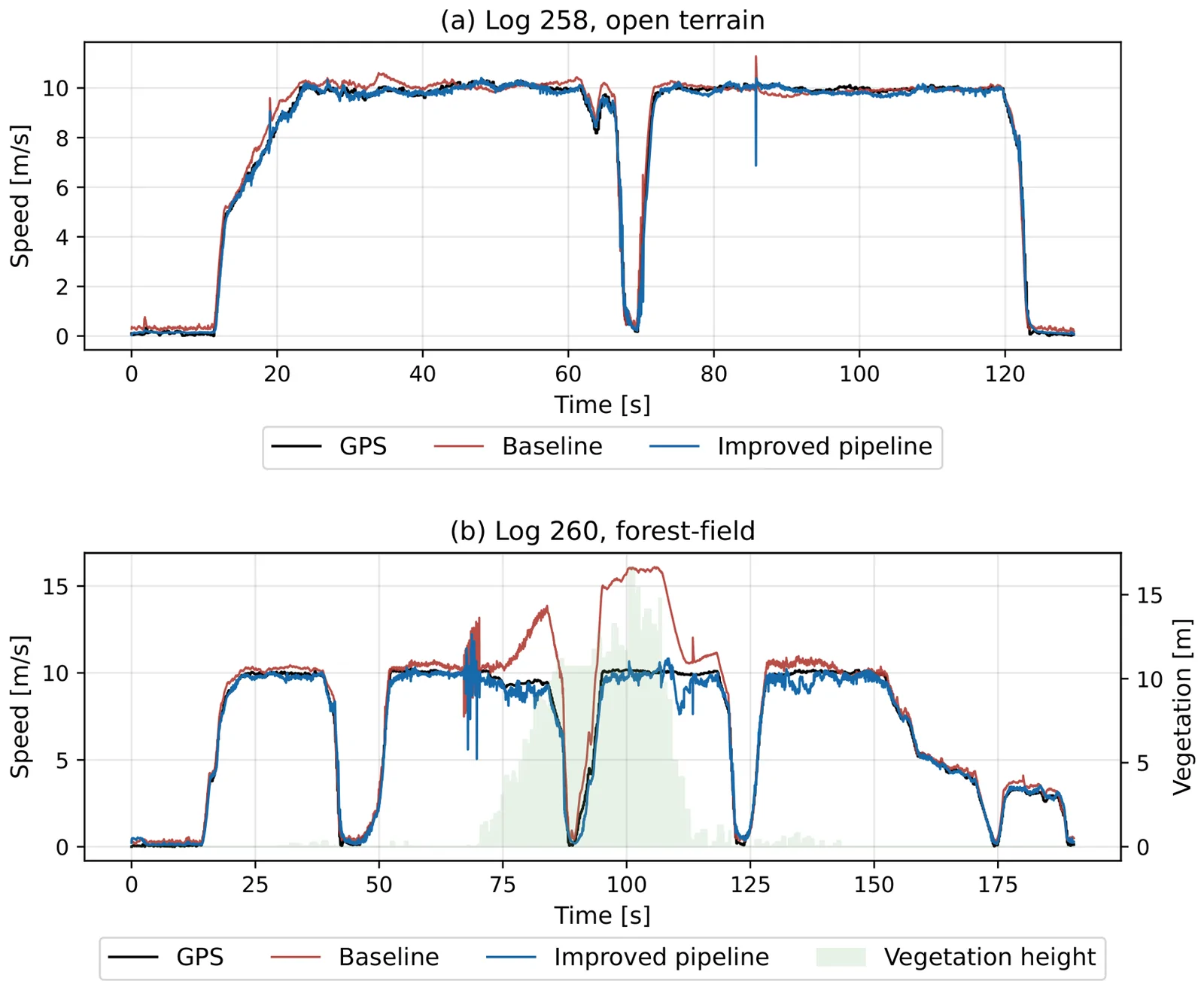
Velocity estimates vs. GPS ground truth for the representative Log 258 and Log 260 flights. (**a**) Log 258, open terrain: baseline vel. MAE =0.25 m/s, improved pipeline =0.13 m/s (−48%). (**b**) Log 260, mixed forest–field: baseline vel. MAE =1.04 m/s, improved pipeline =0.30 m/s (−71%). Black: GPS; red: baseline [3]; blue: improved pipeline. Green shading in (**b**): vegetation height (scaled for display).

Figure 4 shows the velocity time series for Log 260. In the baseline system, velocity spikes of up to 6.1 m/s above GPS ground truth are visible at forest entry and exit transitions. The Kalman innovation gate bounds these abrupt residuals, and the data-driven correction stage reduces the remaining systematic bias during sustained forest overpasses. The final pipeline velocity RMSE on Log 260 is 0.45 m/s compared to 1.86 m/s in the baseline—a 76% reduction.

### 5.9. Dead Reckoning Position Error

**Figure 5:**
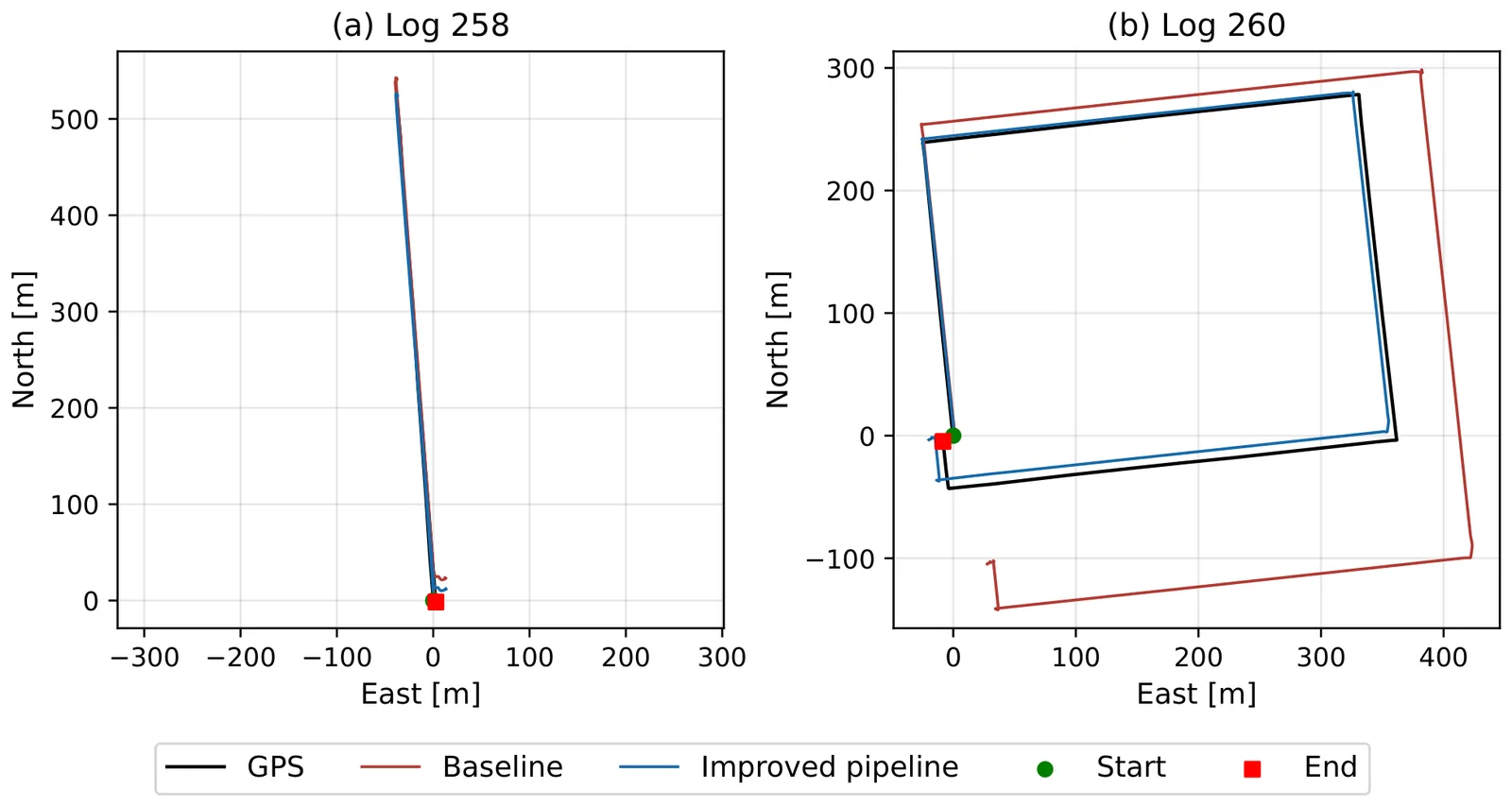
Dead reckoning trajectory vs. GPS ground truth (ENU coordinates, origin at first GPS fix). (**a**) Log 258, open terrain. (**b**) Log 260, forest–field. Black: GPS; red: baseline [3]; blue: improved pipeline. Green circle: start; red square: end. Position MAE reduced from 17.8 m to 7.4 m on Log 258 and from 59.8 m to 7.1 m on Log 260.

**Figure 6:**
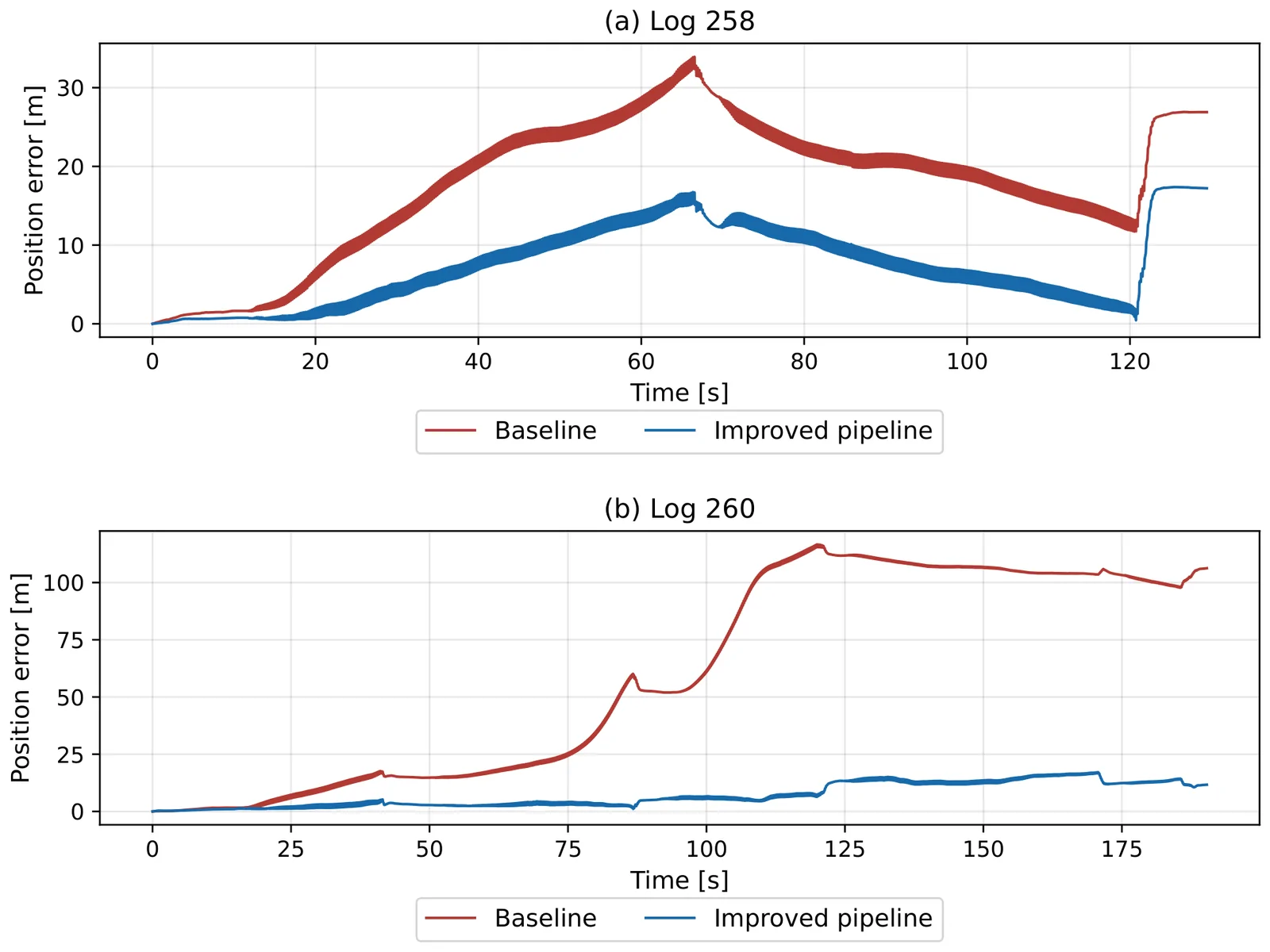
Dead reckoning position error over time. (**a**) Log 258: MAE reduced from 17.8 m to 7.4 m. (**b**) Log 260: MAE reduced from 59.8 m to 7.1 m. The sharp error growth in the baseline on Log 260 around t≈85 s corresponds to the first major forest transition, where an unconstrained velocity spike is integrated into the DR position.

Figure 5 and Figure 6 show the DR trajectories and cumulative position error for the representative Log 258 and Log 260 flights. With the baseline system, DR position error on Log 260 reaches 116.7 m over the 3.2-min flight; the improved pipeline reduces the peak to 17.1 m. The large baseline error on Log 260 relative to Log 258 (116.7 m vs. 33.9 m) stems from the velocity spike at the first major forest transition (t≈85 s), which injects a large erroneous displacement into the dead-reckoning integrator that is never recovered. The innovation gate prevents this injection, which explains the disproportionate improvement on the forest flight.

### 5.10. Computational Cost

Table 9 summarises the per-frame processing time on an Intel Core i7-8750H (Intel Corporation, Santa Clara, CA, USA) at $H_{p}=360$ px, measured as the mean over 1000 frames after GDAL raster warm-up.

## 6. Discussion

The results confirm that the primary source of OF velocity error in mixed-terrain flights is incorrect metric scaling caused by the camera observing the top-of-canopy surface rather than bare ground. On Log 260 (mean vegetation height 2.02 m, maximum 16.65 m at ∼50 m AGL), the baseline velocity MAE reaches 1.04 m/s, substantially above the open-terrain calibration log (0.25 m/s), which has essentially no vegetation. The independent open-terrain validation log remains essentially unchanged, while the mixed forest–field validation log improves from 1.04 to 0.30 m/s velocity MAE. This pattern confirms that the DSM-based AGL correction is the dominant source of improvement for forest terrain rather than a uniform post-processing gain.

The consistent use of DSM (surface model) rather than DTM (terrain model) for AGL is the key design decision. The camera always tracks the highest visible surface; using the bare-earth model introduces a height error equal to the vegetation height, which at 16.65 m canopy and 50 m AGL corresponds to a 33% pixel-scale overestimate. The 5×5 footprint grid averaging additionally stabilises estimates at terrain class boundaries where a single sample might land on a raster artefact or nodata pixel.

This scaling argument follows directly from Equation (1): if s∝h, then a surface-height error δh induces a relative scale and velocity error $\delta s/s\approx \delta h/h_{\mathrm{surf}}$. For Log 260, the largest observed vegetation height is 16.65 m and the surface-relative AGL falls to approximately 42.6 m, so the possible local scale error from using DTM instead of DSM is on the order of 39%. This value is consistent with the large velocity spikes observed at forest transitions and explains why the same correction is not beneficial on Log 259, where DSM–DTM separation remains below 0.16 m.

The disproportionate position improvement on Log 260 (88% vs. 59% on Log 258) is explained by the DR integrator behaviour: the 6.1 m/s velocity spike at the first forest transition (t≈85 s) in the baseline injects a large erroneous displacement that accumulates for the remainder of the flight, pushing the final position error above 116 m. The Kalman innovation gate prevents this injection entirely, and the DR trajectory remains within 17 m of GPS for the full flight duration.

The Kalman gate at γ=4 m/s was chosen to be safely above the maximum legitimate frame-to-frame speed change of a multi-rotor UAV at 30 fps (<2 m/s), while still bounding the observed forest-transition spikes (>6 m/s in the baseline). Implementation parameters for the Kalman filter and correction stage are summarised in Appendix A.

The data-driven correction achieves dimensionless coefficient of determination $R^{2}=0.775$ on the internal held-out split from Log 258, but independent-flight performance is assessed through the complete-pipeline results on Logs 259 and 260. Because Log 258 is nearly flat, the calibration data primarily represent texture-related bias rather than terrain-induced scale error. The residual unexplained variance likely reflects temporal dynamics, intra-scene texture variation, and camera-motion effects that are not fully represented by per-frame descriptors. Future work will therefore examine temporally aware correction and online adaptation using trajectory consistency constraints.

The correction factor should therefore be interpreted as a residual optical flow quality correction rather than as the primary terrain correction. It compensates for effects that are not captured by the pinhole scale model, including texture-dependent OF underestimation, motion blur, small attitude deviations, residual lens distortion, and scene-dependent violations of the single-translation assumption. Potential ways to reduce the need for this learned correction include adaptive OF parameter selection based on coherence, explicit blur detection, feature masking near low-texture regions, robust percentile selection, and replacing Farnebäck OF with more modern dense flow or visual-inertial estimators when sufficient calibration and compute resources are available.

### Generalizability and Operational Envelope

The present validation is geographically limited to one Polish test site and to three logs. Consequently, the results should be interpreted as evidence for the proposed error mechanism and processing strategy rather than as a complete multi-region robustness assessment. Deployment in different geographic regions requires locally available, co-registered DTM/DSM rasters in an appropriate coordinate reference system. The geometric part of the method is transferable after camera intrinsic parameters, image resolution, frame rate, and mounting orientation are updated; however, the data-driven correction module should be recalibrated when the UAV platform, camera, lens, stabilisation behaviour, or image-processing pipeline changes.

The sensitivity to footprint grid size and raster resolution was not exhaustively evaluated in the current dataset. All reported experiments use a 5×5 footprint grid and 1 m GSD terrain rasters. The expected sensitivity is governed by the ratio between raster resolution, camera footprint size, and terrain discontinuity scale. A finer grid reduces single-sample boundary artefacts but increases the influence of raster noise, while a coarser raster can smooth canopy edges and attenuate the DSM–DTM height difference that the method is designed to exploit. Seasonal vegetation changes are another source of uncertainty: if the DSM is acquired under leaf-on conditions but the flight occurs under leaf-off conditions, or vice versa, the surface model may misrepresent the visible canopy height. A surface-height error δh produces an approximate relative velocity-scale error of $\delta h/h_{\mathrm{surf}}$; for example, a 5 m canopy-height mismatch at 50 m surface-relative altitude corresponds to approximately 10% scale error.

Adverse visual conditions such as low illumination, rain, fog, and strong motion blur were not present in the evaluated logs. These conditions are expected to reduce optical flow coherence and increase texture-dependent bias and therefore require separate validation before operational use. Similarly, no same-dataset comparison with modern visual-inertial odometry or learning-based navigation systems is reported, because the available logs do not include the camera calibration, synchronisation, and raw inertial streams required for a fair VIO benchmark. The proposed pipeline should therefore be viewed as a terrain-aware optical flow/dead-reckoning correction method, not as a replacement for tightly coupled VIO or learned SLAM systems when those systems can be deployed with complete calibration and compute resources.

**Limitations.** The DTM/DSM rasters cover Poland (EPSG:2180) and must be re-acquired for other regions. Processing runs at ∼18 ms/frame on a desktop CPU, which exceeds real-time at 30 fps (∼33 ms budget); GPU acceleration of Farnebäck or reducing $H_{p}$ below 360 px would close this gap. The data-driven correction currently requires GPS-labelled calibration data and cannot adapt in-flight without a reference signal. The validation set was expanded to three logs, but it remains limited to 7.3 min of daytime, clear-sky operation at a single geographic site; performance under low illumination, rain, and substantially different vegetation structure has not yet been evaluated.

## 7. Conclusions

This paper presented three improvements to an optical flow-based velocity estimation pipeline for GPS-denied UAV navigation, building on the fusion architecture first introduced in [3]. Camera footprint area mean sampling from co-registered DTM/DSM rasters eliminates the dominant scale bias over forested terrain. A Kalman filter with innovation gate suppresses boundary-transition spikes. A lightweight data-driven correction stage based on per-frame flow, texture, and terrain descriptors captures residual scene-dependent bias.

Validation on three real PX4 flight logs demonstrates terrain-dependent behaviour. On open terrain, Log 258 improves after calibration whereas the independent Log 259 remains essentially unchanged, as expected when DTM and DSM elevations are nearly identical. On the mixed forest–field validation flight (Log 260, max vegetation height 16.65 m), velocity MAE decreases from 1.04 m/s to 0.30 m/s (−71%) and position MAE from 59.8 m to 7.1 m (−88%). The results confirm that the terrain-aware DSM-based AGL correction is the dominant contributor particularly in vegetated environments, while broader robustness must be verified on larger multi-site datasets.

All three additions impose negligible computational overhead beyond the Farnebäck computation (<3 ms combined at $H_{p}=360$ px), indicating that the approach is compatible with practical post-processing workflows and may be adapted for real-time implementation after further engineering optimisation.

## Figures and Tables

**Table 1:** Camera and video parameters used by the processing model.

Parameter	Value/Treatment
UAV/camera platform	Yuneec H520 (Yuneec International, Kunshan, Jiangsu, China), nadir-facing gimbal-stabilised video
Frame size used	1920 × 1080 px
Frame rate	30 fps
Diagonal field of view	91°
Projection model	Equivalent pinhole model expressed through diagonal FOV
Pixel pitch and focal length	Not used independently; absorbed into the FOV-based scale model
Exposure/shutter metadata	Not available in exported logs; motion-blur effects assessed indirectly by OF coherence

**Table 2:** Output CSV column definitions.

Column	Description
frame_number	Sequential video frame index
speed_mps	OF-derived ground speed after Kalman and data-driven correction (m/s)
altitude	Barometric altitude AGL from take-off (m)
heading	Local-frame track angle, [0°, 360°); column name retained for CSV compatibility
dr_lat	Dead reckoning latitude estimate (deg)
dr_lon	Dead reckoning longitude estimate (deg)
gps_lat	GPS latitude ground truth (deg)
gps_lon	GPS longitude ground truth (deg)
gps_vel	GPS speed over ground (m/s)
dr_east	Dead reckoning East displacement from origin (m)
dr_north	Dead reckoning North displacement from origin (m)
dr_up	Dead reckoning Up displacement from origin (m)
agl_used	AGL altitude used for OF scaling (=agl_surface)
agl_ground	AGL above bare-earth DTM (m)
agl_surface	AGL above DSM surface (m)
terrain_model	Source tag: “surface” or “alt_only”
of_p10…of_p90	Flow magnitude percentiles converted to m/s
of_coherence	Directional coherence C∈[0,1]
texture_std	Grayscale frame standard deviation

**Table 3:** Farnebäck parameterisation used in the experimental evaluation.

Parameter	Value	Meaning
pyr_scale	0.5	Scale factor between pyramid levels
levels	5	Number of pyramid levels
winsize	13	Averaging window size (px)
iterations	3	Iterations per pyramid level
poly_n	5	Pixel neighbourhood for polynomial expansion
poly_sigma	1.1	Gaussian std for polynomial smoothing
flags	0	Standard mode

**Table 4:** Height quantities used in terrain-aware scaling.

Symbol	Meaning
$z_{\mathrm{baro}}$	Barometric altitude above take-off, from PX4 local-position logs
${\bar{h}}_{\mathrm{DTM}}$	Area mean bare-earth elevation under the camera footprint
${\bar{h}}_{\mathrm{DSM}}$	Area mean visible surface elevation under the camera footprint
$h_{\mathrm{gnd}}$	UAV height above bare earth, used only to estimate vegetation height
$h_{\mathrm{surf}}$	UAV height above the visible surface, used for OF metric scaling
$\Delta h_{\mathrm{veg}}$	Estimated vegetation/building height, $h_{\mathrm{gnd}}-h_{\mathrm{surf}}$

**Table 5:** Flight log characteristics.

Log	Duration	Frames	Mean Surface AGL	Surface AGL Range	Max Veg. Height
258 (open terrain; calibration)	∼2.2 min	3884	61.2 m	60.0–61.8 m	0.21 m
259 (open terrain; validation)	∼2.0 min	3618	120.3 m	119.5–121.3 m	0.16 m
260 (forest–field; validation)	∼3.2 min	5711	57.6 m	42.6–60.2 m	16.65 m

Vegetation height $=h_{\mathrm{gnd}}-h_{\mathrm{surf}}$ from DTM/DSM area mean sampling. Surface AGL is the altitude above the visible surface used in Equation (4). GPS velocity ground truth at ∼5 Hz. Log 258 used for calibration; Logs 259–260 held out for independent evaluation.

**Table 6:** Velocity and position error comparison: baseline [3] vs. improved pipeline. Velocity error computed on frames with $v_{\mathrm{GPS}}>0.1$ m/s. MAE denotes mean absolute error and RMSE denotes root mean square error. Position error is the Euclidean DR displacement error with respect to GPS.

Log	System	Vel. MAE [m/s]	Vel. RMSE [m/s]	Pos. MAE [m]	Pos. RMSE [m]
258 (open)	Baseline [3]	0.251	0.335	17.82	19.80
	Improved pipeline	**0.131**	**0.215**	**7.38**	**8.92**
	Improvement	−47.9%	−35.8%	−58.6%	−54.9%
259 (open, held-out)	Baseline [3]	**1.202**	**1.266**	**38.70**	**47.12**
	Improved pipeline	1.217	1.281	38.70	47.16
	Change	+1.2%	+1.2%	+0.0%	+0.1%
260 (forest, held-out)	Baseline [3]	1.043	1.862	59.83	74.42
	Improved pipeline	**0.300**	**0.450**	**7.06**	**8.78**
	Improvement	−71.3%	−75.8%	−88.2%	−88.2%

Bold: lower (better) values. Log 258 and Log 259: mostly flat open terrain. Log 260: mixed agricultural–forest terrain (mean vegetation height 2.02 m, max 16.65 m).

**Table 7:** Paired sign-test summary for frame-wise absolute velocity errors. Positive differences indicate lower error for the improved pipeline.

Log	Frames	Improved Frames	Mean Error Reduction	Sign-Test *p*
			[m/s]	
258	3575	2553	0.120	<10^−100^
259	3450	646	−0.015	<10^−100^
260	5274	4017	0.744	<10^−100^

The negative value on Log 259 confirms that the terrain-aware pipeline does not create a uniform apparent improvement on flat open terrain; this log is retained as an independent control case rather than as a positive result.

**Table 8:** Directional coherence statistics across the representative calibration and forest–field validation logs.

Log	Mean *C*	Std *C*	Frames	Terrain
258	0.871	0.266	3884	Open/flat
260	0.846	0.246	5711	Forest–field mix

**Table 9:** Per-frame processing time breakdown ($H_{p}=360$ px, Core i7-8750H).

Component	Time [ms]
Frame resize to $H_{p}=360$ px	<0.5
Farnebäck OF (CPU, 5 pyramid levels)	∼15
Magnitude sort + percentile extraction	∼2
Terrain 5×5 footprint (GDAL, hot)	∼1
Kalman filter update	<0.1
Data-driven correction stage	<0.1
CSV write + console output	<0.5
**Total per frame**	∼**18**

Bold denotes the total per-frame processing time, summed over all component stages. Farnebäck computation accounts for >80% of total time. All terrain, Kalman, and correction-stage overhead is negligible relative to OF.

## Data Availability

The flight logs and derived evaluation data supporting the findings of this study are available from the corresponding author upon reasonable request, subject to institutional and operational constraints. The implementation used in this study is not publicly released at this stage due to ongoing development and planned technology transfer.

## Abbreviations

The following abbreviations are used in this manuscript:

AGL	Above Ground Level
CRS	Coordinate Reference System
CSV	Comma-Separated Values
DR	Dead Reckoning
DSM	Digital Surface Model
DTM	Digital Terrain Model
ENU	East-North-Up (local coordinate frame)
FOV	Field of View
GDAL	Geospatial Data Abstraction Library
GPS	Global Positioning System
GSD	Ground Sample Distance
IMU	Inertial Measurement Unit
MAE	Mean Absolute Error
NED	North-East-Down (local navigation frame convention)
OF	Optical Flow
PX4	Open-source flight controller autopilot
$R^{2}$	Coefficient of determination (dimensionless)
RMSE	Root Mean Square Error
SBC	Single Board Computer
UAV	Unmanned Aerial Vehicle
WGS-84	World Geodetic System 1984

## Appendix A. Implementation Parameters

Table A1 summarises the parameters required to reproduce the reported pipeline configuration. These values were fixed before running the independent validation logs.

**Table A1:** Implementation parameters used in the reported experiments.

Parameter	Value
Processing height $H_{p}$	360 px
Frame skip *S*	1
Footprint sampling grid	5×5
Kalman process noise *q*	0.01 (m/s)^2^/frame
Kalman measurement noise *r*	2.0 (m/s)^2^
Kalman innovation gate γ	4.0 m/s
MLP hidden layers	64–32–16
MLP activation	ReLU
MLP optimiser	Adam
MLP $L_{2}$ penalty	$10^{-4}$
MLP learning rate	$10^{-3}$
MLP maximum epochs	500
MLP early-stopping validation fraction	0.10
MLP random seed	42
Correction-factor projection interval	[0.5,1.5]
